# Optimized Effects of Fisetin and Hydroxychloroquine on ER Stress and Autophagy in Nonalcoholic Fatty Pancreas Disease in Mice

**DOI:** 10.1155/jdr/2795127

**Published:** 2025-04-14

**Authors:** Mahboobe Sattari, Amin Karimpour, Maryam Akhavan Taheri, Bagher Larijani, Reza Meshkani, Ozra Tabatabaei-Malazy, Ghodratollah Panahi

**Affiliations:** ^1^Endocrinology and Metabolism Research Center, Endocrinology and Metabolism Clinical Sciences Institute, Tehran University of Medical Sciences, Tehran, Iran; ^2^Department of Clinical Biochemistry, Faculty of Medicine, Tehran University of Medical Sciences, Tehran, Iran; ^3^Students' Scientific Research Center (SSRC), Tehran University of Medical Sciences, Tehran, Iran; ^4^Anatomical Sciences Research Center, Institute for Basic Sciences, Kashan University of Medical Sciences, Kashan, Iran; ^5^Non-Communicable Diseases Research Center, Endocrinology and Metabolism Population Sciences Institute, Tehran University of Medical Sciences, Tehran, Iran

**Keywords:** autophagy, ER stress, fatty pancreas, fisetin, hydroxychloroquine, metabolic syndrome

## Abstract

**Background:** Fat accumulation in the pancreas, known as nonalcoholic fatty pancreatic disease (NAFPD), is associated with obesity and may lead to prediabetes and Type 2 diabetes. Reducing endoplasmic reticulum stress and enhancing autophagy could offer therapeutic benefits. This study examines the effects of fisetin (FSN) and hydroxychloroquine (HCQ) on NAFPD.

**Method:** Forty-eight Male C57BL/6 J mice were assigned to a standard chow diet (SCD) or a high-fat diet (HFD) for 16 weeks. The HFD group was divided into five subgroups; each group contains eight mice: HFD, HFD + V (vehicle), HFD + FSN, HFD + HCQ, and HFD + FSN + HCQ. FSN was given daily at 80 mg/kg, and HCQ was injected IP at 50 mg/kg twice weekly for more 8 weeks. Insulin resistance was assessed through OGTT and HOMA-IR. Histological analysis of pancreatic tissue was conducted, and the protein and mRNA levels of molecules associated with ER stress and autophagy were assessed using PCR and immunoblotting techniques.

**Result:** FSN and HCQ significantly reduced weight gain, pancreatic adipocyte accumulation, and insulin resistance caused by HFD in obese mice, with the combination of the two compounds producing even more pronounced effects. Additionally, the HFD increased the expression of UPR markers ATF4 and CHOP, a response that was further intensified by HCQ. In contrast, FSN attenuated the UPR by regulating GRP78 levels. Furthermore, the HFD resulted in a significant decrease in the LC3II/LC3I ratio and an accumulation of p62 protein due to reduced p-AMPK levels. Following treatment with FSN, these alterations were reversed, leading to decreased mTOR expression and increased levels of autophagy markers such as ATG5 and Beclin1.

**Conclusion:** Our study reveals that FSN and HCQ effectively combat HFD-induced NAFPD, improving insulin sensitivity and addressing pancreatic fat deposition linked to metabolic syndrome. While HCQ may cause endoplasmic reticulum stress, FSN offers protective effects, supporting their combined use for better treatment outcomes.

## 1. Introduction

Obesity is implicated in various metabolic derangements that elevate the predisposition to metabolic syndrome, Type 2 diabetes (T2DM), and cardiovascular disorders. These effects stem from the excessive deposition of fat in vital organs such as the liver, heart, muscles, and kidneys [1]. Notably, fat accumulation in the pancreas, termed as nonalcoholic fatty pancreatic disease (NAFPD), is recognized in the context of obesity and metabolic disorders [2]. Emerging evidence from human studies suggests a potential link between NAFPD and the pathogenesis of prediabetes and T2DM, attributed to the adverse impact of pancreatic fat accumulation on *β*-cell function and carbohydrate metabolism. Moreover, correlations have been observed between NAFPD and markers of atherosclerosis, such as carotid intima–media thickness, underscoring its association with cardiovascular diseases. Alarmingly, NAFPD is also associated with an elevated risk of pancreatic cancer [3], highlighting the importance of early detection given the limited treatment options for this malignancy [4]. Despite its significance, NAFPD remains a frequently overlooked condition, with its prevalence mirroring the rising rates of obesity. Age-related increases in pancreatic fat content further compound this issue [5]. A meta-analysis revealed a substantial prevalence of NAFPD (33%), irrespective of age and gender, with healthy individuals exhibiting pancreatic fat levels averaging 4.48%. The identification of a threshold value of 6.2% for NAFPD underscores the clinical relevance of quantifying pancreatic fat content [6].

The pathophysiological mechanisms underlying pancreatic fat accumulation remain incompletely understood. Existing evidence highlights the detrimental impact of pancreatic fat on endocrine function, triggering a harmful cycle of increased fat deposition and impaired *β*-cell function. Consequently, there is a rising prevalence of NAFPD among individuals with T2DM [7] and an increasing occurrence of T2DM among those with fatty pancreas [8]. Hyperglycemia exacerbates intracellular triglyceride accumulation by inhibiting enzymes crucial for mitochondrial *β*-oxidation. Moreover, factors like adipose-derived cytokines, elevated levels of free fatty acids, and hyperglycemia contribute to *β*-cell destruction and subsequent replacement by adipocytes within the pancreatic tissue [9]. The endoplasmic reticulum (ER) plays a pivotal role in proinsulin folding, highlighting the importance of ER proteostasis for *β*-cell function and survival in both physiological and pathological conditions. Intracellular Ca^2+^ handling by the ER is crucial for insulin secretion [10]. Disruption of this delicate balance by environmental factors like a high-fat diet (HFD) can trigger the unfolded protein response (UPR) [11]. The UPR is governed by three sensors that typically bind to 78 kDa glucose-regulated protein (GRP78); however, under ER stress conditions, these sensors activate upon dissociation from GRP78. This activation leads to the transcription of factors such as activating transcription Factor 4 (ATF4), which in turn activates downstream UPR genes like C/EBP homologous protein (CHOP). Ultimately, this cellular response initiates a series of events to manage the degradation of misfolded proteins, potentially culminating in apoptosis. Given that severe ER stress can provoke inflammation and *β*-cell apoptosis, mitigating ER stress could offer a promising therapeutic avenue for NAFPD treatment [12].

Autophagy is the cellular mechanism utilized in stressful conditions to counteract the negative effects of ER stress. It plays a crucial role in maintaining cellular homeostasis by mitigating ER stress, particularly through selective autophagy known as ER-phagy [13]. In times of ER stress, all three branches of the UPR can stimulate autophagy and the formation of autophagosomes. This activation is achieved by upregulating the expression of various autophagy-related protein (ATG) genes, suppressing autophagy inhibitors, and regulating key kinases like AMPK and mechanistic target of rapamycin (mTOR). The process of autophagy commences with AMPK phosphorylation, followed by Beclin-1-mediated phagophore nucleation. Phagophore elongation is facilitated by two systems (ATG5-based and LC3II-based) leading to autophagosome formation, which then fuses with lysosomes for cargo degradation. mTOR acts as a sensor for nutrient availability and can inhibit autophagy. Autophagy serves as an adaptive mechanism in response to cellular damage, enabling the removal of impaired organelles and misfolded proteins [14]. Decreased autophagy renders cells vulnerable to stress-induced cell death. Research indicates that short-term exposure (8 weeks) to a HFD enhances autophagic flux in pancreatic *β*-cells, protecting against terminal ER stress induction [15]. However, prolonged HFD exposure (16 weeks) diminishes autophagy efficiency, posing challenges to insulin secretion [16]. The upregulation of autophagy may offer a promising target for therapeutic interventions.

Considering the potential adverse effects of conventional medical treatments, the study of complementary treatments that are natural and safe products, such as herbal medicines or their active ingredients, is of interest and focus. Fisetin (FSN), 3,3⁣′,4⁣′,7-tetrahydroxy flavone, is present in various types of plants [17], which has shown in various studies that it can have favorable effects in the management of metabolic disorders [18]. This is attributed to its ability to regulate energy metabolism, deal with inflammation, and control oxidative stress [19]. For example, recent studies indicate that FSN can protect against HFD-induced metabolic disorders, including the prevention of hepatic steatosis and obesity, by limiting lipogenesis and stimulating lipolysis in the liver and white adipose tissue, respectively. Additionally, FSN increased the weight of brown adipose tissue and skeletal muscles, stimulated thermogenic gene expression in BAT, and enhanced tricarboxylic acid cycle gene expression in skeletal muscle [20]. FSN can regulate insulin signaling by increasing IRS expression and involving the PI3K/AKT signaling pathway [21]. FSN can reduce liver inflammation and fat deposition through TNF-*α*/RIPK3 signaling pathway [22]. FSN has also been shown to suppress the inflammatory response induced by ER stress and help to ameliorate cardiac damage induced by metabolic stimuli [23].

Although the protective effects of FSN against metabolic stress have been reported, questions still remain regarding the underlying molecular mechanisms and possible interactions between them. Specifically, no studies have been conducted on the effect of FSN on the autophagy process and its secondary effects on other underlying pathways in the pathogenesis of NAFPD. In this study, we employed hydroxychloroquine (HCQ) alongside FSN primarily as an autophagy inhibitor to delineate the contribution of the autophagy pathway to FSN's effects on NAFPD. We aim to determine whether the beneficial effects of FSN persist even with the inhibition of autophagy. This approach will enhance our understanding of the underlying molecular mechanisms elicited by FSN. Furthermore, HCQ has been utilized as a treatment for autoimmune diseases in humans, and several studies involving these patients suggest that HCQ has favorable effects on metabolic aspects [24, 25]. However, despite these promising clinical findings, ambiguities regarding its mechanisms of action remain, which may be addressed through animal studies. Given that certain long-term side effects of HCQ have been reported, exploring combination therapies could help mitigate these effects. If FSN and HCQ show effectiveness in these metabolic processes, they may hold potential as therapeutic or preventive options for NAFPD.

## 2. Materials and Methods

### 2.1. Chemicals and Drugs

FSN, with a CAS No. and HPLC purity of ≥ 98%, Batch No. 528-48-3, was sold by Henan Tianfu Chemical Co., based in Henan, China, under the label TF20220119. HCQ, with CAS Number 747-36-4, was supplied by Iran Chemical Ph. under Serial No. HCS0040299, through Raw Materials Corporation in Tehran, Iran. The primary antibodies used in western blot analysis were LC3B (#2775 Cell Signaling), p62 (sc-10117, Santa Cruz, California), p-AMPK*α*1/2 (sc-33524, Santa Cruz, California), AMPK*α*1/2 (sc-74461, Santa Cruz, California), and *β*-actin (sc-517582, Santa Cruz, California). The secondary antibodies used in western blot analysis were the mouse anti-rabbit IgG-HRP antibody (sc-2357, Santa Cruz, California) and m-IgG*κ*BP-HRP (sc-516102, Santa Cruz, California).

### 2.2. Animals

Male C57BL/6 J mice aged 7–8 weeks and weighing 18–20 g were acquired from the Iranian Pasteur Institute. They were housed in a controlled environment with a standard 12-h light/12-h dark cycle, consistent temperature of 23°C–25°C, and humidity ranging from 50% to 60%. The mice had access to pathogen-free food and water at all times and were provided with enrichment materials such as shredded paper and plastic tubes in their cages. Daily inspections and weekly cleanings were carried out. The protocols for animal experiments were approved by the Tehran University of Medical Science's Research Ethics Committees of Laboratory Animals (IR.TUMS.AEC.1402.063).

### 2.3. Experimental Protocol

After a 2-week adaptation period, 48 mice were randomly assigned to one of two diets: the standard chow diet (SCD) (10% kcal fat; D12450B, Open Source Diets) (*n* = 8) or the HFD (60% kcal fat; D12492, open source diets, research diets) (*n* = 40). After 16 weeks, five groups (*n* = 8) were randomly selected from the HFD group: HFD, HFD + Vehicle (V), HFD + FSN, HFD + HCQ, and HFD + FSN + HCQ. FSN was administered (80 mg/kg) daily via gavage alongside the HFD, while HCQ was injected (50 mg/kg) intraperitoneally twice a week. Dosages were adjusted based on weekly body weight.

### 2.4. Glucose Tolerance Test

The OGTT was conducted to evaluate metabolic tolerance in the last week of the treatment phase [26]. The test included a 10-h overnight fast, followed by the administration of a 10% *α*-D-glucose solution via gavage at a dose of 2 g/kg. Blood glucose levels were monitored at 15, 30, 60, and 120-min intervals using a glucometer (On Call Plus) to analyze blood drops from the mice's tail.

### 2.5. Sampling

At the conclusion of the treatment period, the mice underwent a 10-h fasting period before they were euthanized using CO_2_ asphyxiation and decapitation. Approximately 0.6 mL of blood was collected into a tube and allowed to clot at room temperature for 20 min. The blood sample was then centrifuged, and the resulting supernatant was analyzed for biochemical parameters. Afterwards, the pancreas tissues were carefully removed, weighed, and sectioned. One portion of the tissues was preserved in a tube containing a 4% formalin, while the rest were frozen in liquid nitrogen and stored at −80°C for future analysis using western blot and qRT-PCR techniques.

### 2.6. Biochemical Parameters Analysis

The fasting plasma levels of glucose were measured using the CL2000i Autoanalyzer. Insulin plasma concentration was determined using a commercial ELISA kit (Mouse Insulin ELISA Kit, ZellBio GmbH, Cat. No: ZB-10062C-M9648, Germany). The HOMA-IR value was calculated using the formula HOMA − IR = (glucose in mg/dL) × (insulin in mU/L)/405. The insulin-to-protein concentration ratio was established by measuring protein levels using the Bradford assay and insulin levels using the chemiluminescence method in the pancreatic lysates.

### 2.7. Pancreas Histological Analysis

Pancreas tissue specimens were preserved for a minimum of 24 h in paraformaldehyde. Following a graded series of ethanol dehydration, the specimens were cleaned in xylene and embedded in paraffin wax. Using a rotary microtome, tissue blocks were sectioned at a 5-*μ*m thickness. Hematoxylin and eosin were used to stain the sections. Histological features were viewed using an Olympus light microscope (Olympus, Tokyo, Japan) by a qualified pathologist. Histopathological alterations in the pancreas of HFD groups were examined in comparison to SCD groups, focusing on interlobular adipocyte infiltration, interstitial edema, and inflammation.

### 2.8. RNA Extraction and Quantitative Real-Time PCR

Total RNA was isolated from fresh-frozen pancreas tissues using the AnaCell Super RNA Extraction Kit in Tehran, Iran. The quality and concentration of the extracted RNA were assessed using a NanoDrop instrument (Thermo Scientific NanoDrop OneC). Subsequently, 2000 ng of the extracted RNA was reverse transcribed into cDNA using a cDNA synthesis kit from AnaCell. The mRNA levels of key molecules involved in UPR regulation (such as GRP78, ATF4, and CHOP) and autophagy regulation (including mTOR, ATG6/Beclin-1, ATG5, and p62/SQSTM1) in the pancreas were quantified using qPCR Master Mix from AnaCell on a real-time PCR cycler (Applied Biosystems StepOne RT PCR machine). Details of the primers used for measuring mRNA expression levels in Real-Time PCR can be found in Table S1 of the Supporting Information section.

### 2.9. SDS-PAGE and Western-Blot Analysis

Mouse pancreas tissues were homogenized using lysis buffer. Following centrifugation, the protein concentrations in the supernatant were determined using the Bradford method [27]. Bovine serum albumin served as the reference protein. Equal amounts of protein lysate were loaded onto a 10%–12% SDS-PAGE gel and transferred to a PVDF membrane for protein blotting. Following blockage with 2% nonfat dry milk in TBS-T, the membranes were incubated with primary antibodies overnight at 4°C. Subsequently, secondary antibodies were used for probing at room temperature. Each lane on the gel contained a pooled standard sample, and ECL-advanced reagents were employed for band identification. *β*-Actin served as a loading control, and densitometric analysis was performed using ImageJ.

### 2.10. Statistical Analyses

The results are presented as mean ± SEM. Statistical analysis included one-way ANOVA followed by Tukey's multiple comparison test. Statistical significance was defined as *p* < 0.05, with significance levels denoted as ∗*p* < 0.05, ∗∗*p* < 0.01, ∗∗∗*p* < 0.001, and ∗∗∗∗*p* < 0.0001. GraphPad Prism 9 from San Diego, California, was used for statistical analysis.

## 3. Results

### 3.1. Improvement of HFD-Induced Obesity and NAFPD in Mice by FSN and HCQ

**Figure 1:**
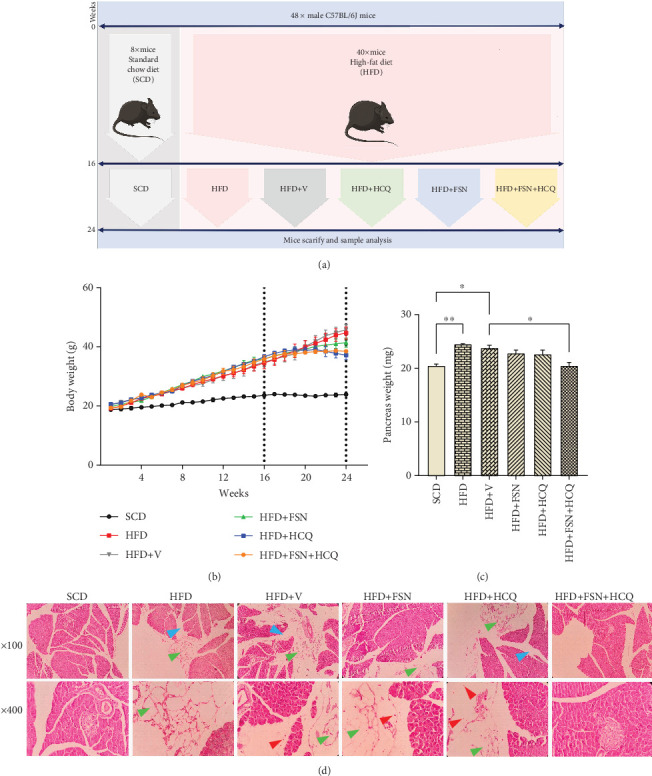
Improvement of HFD-induced obesity and NAFPD in mice by fisetin and hydroxychloroquine. (a) Schematic diagram of the experimental procedure. (b) Change of body weight during a 24-week study period. (c) The pancreas weight (milligrams). (d) The representative histological images of H&E-stained sections (100× magnification in up and 400× magnification in down). A 24-week period of HFD resulted in interlobular adipocyte infiltration (which showed by green arrows), interstitial edema (enlarged interlobular space area) (which showed by blue arrows), as well as inflammation indicated by the presence of inflammatory cells (which showed by red arrows) in pancreatic tissue of HFD and HFD + V mice compared to SCD mice. However, HFD + FSN and HFD + HCQ groups showed a moderate improvement, and HFD + FSN + HCQ mice exhibited a noticeable change near the SCD histology (*n* = 7). Values are presented as means ± SEM. ∗∗∗∗*p* < 0.0001, ∗∗∗*p* < 0.001, ∗∗*p* < 0.01, ∗*p* < 0.05. NAFPD, nonalcoholic fatty pancreatic disease; HFD, high-fat diet; V, vehicle; SCD, standard chow diet; FSN, fisetin; HCQ, hydroxychloroquine; H&E, hematoxylin and eosin.

We fed HFD to male C57BL/6 mice for 16 weeks in order to induce obesity and NAFPD (Figure 1a). During the 16 weeks of experiment, the HFD-fed mice's body weight increased considerably more than that of the SCD group. After 8 weeks of drugs treatment, FSN, FSN + HCQ, and more notably HCQ hindered the weight gain in contrast to HFD mice (Figure 1b). Additionally, the HFD (*p* < 0.01) and HFD + V (*p* < 0.05) groups were observed to have considerably greater pancreatic mass expansion compared with the SCD groups (Figure 1c). Notably, neither of the two treatment groups, HFD + FSN and HFD + HCQ, exhibited significant differences compared to the HFD + V group, indicating that they similarly affected pancreatic weight. However, in contrast to the HFD + V group, the pancreatic weight in the HFD + FSN + HCQ treatment group was significantly lower (*p* < 0.05). Additionally, analysis of pancreas morphology using H&E staining revealed that a 24-week period of a HFD resulted in a range of pancreatic tissue alterations in obese mice compared to nonobese control mice, such as, interlobular adipocytes infiltration, interstitial edema, and, inflammation (presence of inflammatory cells). However, treatment with FSN and HCQ showed improvement, with the most significant changes observed in mice receiving both FSN and HCQ together (Figure 1d).

### 3.2. Restoration of Glucose Homeostasis and Insulin Resistance by FSN and HCQ in HFD Mice

**Figure 2:**
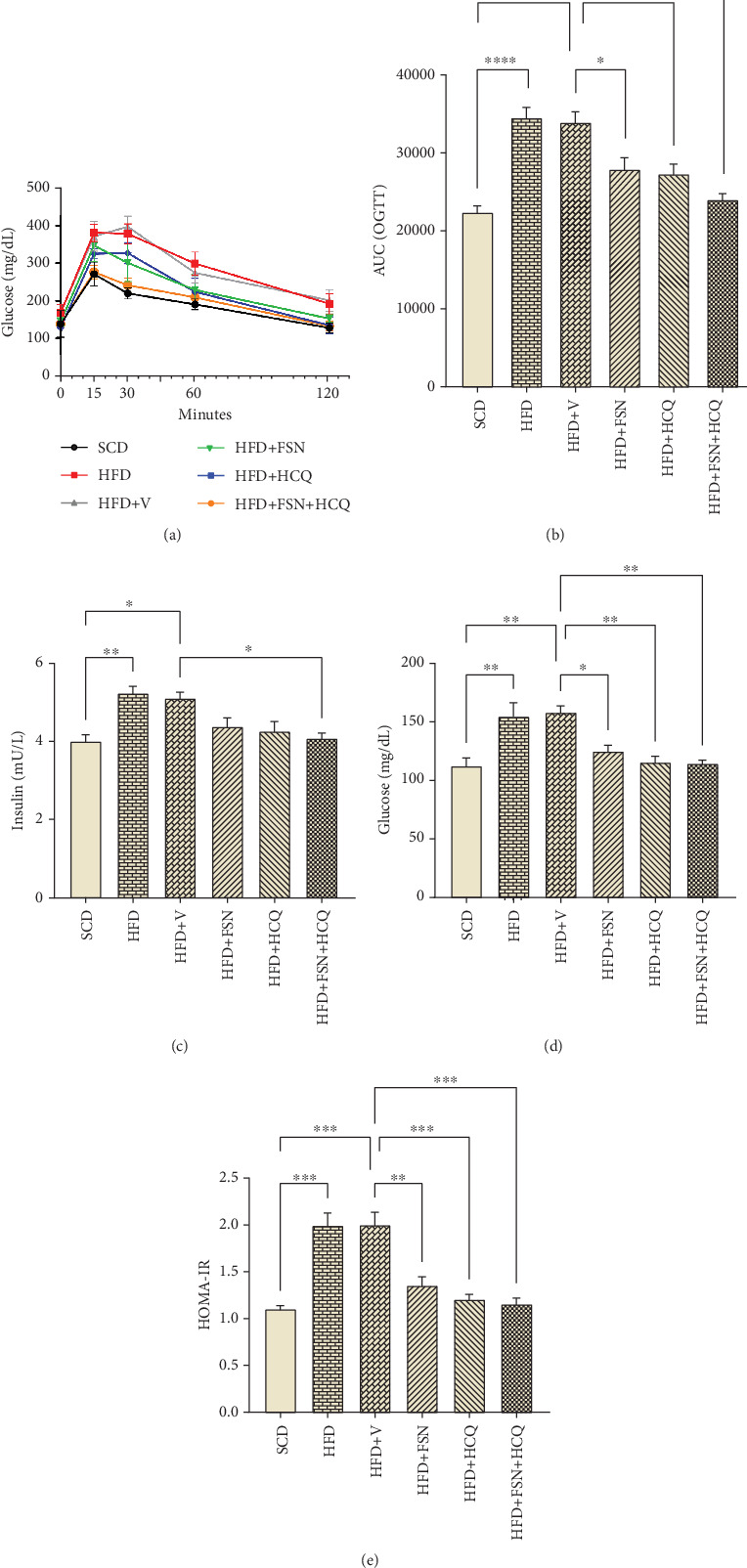
Restoration of glucose homeostasis and insulin resistance by fisetin and hydroxychloroquine in HFD mice. (a) OGTT, (b) AUC of blood glucose level during OGTT, Serum (c) insulin and (d) glucose in mice after 10 h of fasting, (e) HOMA-IR (*n* = 5). Values are presented as means ± SEM. ∗∗∗∗*p* < 0.0001, ∗∗∗*p* < 0.001, ∗∗*p* < 0.01, ∗*p* < 0.05. HFD, high-fat diet; V, vehicle; SCD, standard chow diet; FSN, fisetin; HCQ, hydroxychloroquine; OGTT, oral glucose tolerance test; AUC, area under the curve; HOMA-IR, homeostasis model assessment of insulin resistance.

The analysis of the area under the curve (AUC) of OGTT showed that HFD (*p* < 0.0001) and HFD + V (*p* < 0.0001) mice exhibited challenged glucose homeostasis. Nonetheless, treatment with FSN (*p* < 0.05) and HCQ (*p* < 0.05) resulted in notable improvements, with the most significant changes occurring in mice that received both FSN and HCQ in conjunction (*p* < 0.001) (Figure 2a,b). Fasting serum insulin levels rose in HFD (*p* < 0.01) and HFD + V (*p* < 0.05) groups, which indicated more severe insulin resistance state. Moreover, treatment with FSN and HCQ reduced these levels by 13% and 16%, respectively. Notably, the combination of FSN and HCQ led to a significant reduction (*p* < 0.05) of 20% (Figure 2c). Fasting serum glucose was increased in the HFD (*p* < 0.01) and HFD + V (*p* < 0.01) group, while FSN (*p* < 0.05), HCQ (*p* < 0.01), and FSN + HCQ (*p* < 0.01) treatment strategies caused a significant decrease (Figure 2d). Collectively, mice showed approximately a double HOMA-IR index after HFD (*p* < 0.001) and HFD + V (*p* < 0.001), while FSN (*p* < 0.01), HCQ (*p* < 0.001), and FSN + HCQ (*p* < 0.001) treatments reduced it significantly (Figure 2e).

### 3.3. Reinforcement of HFD-Induced ER Stress by HCQ and Inhibition by FSN in Mouse Pancreas

**Figure 3:**
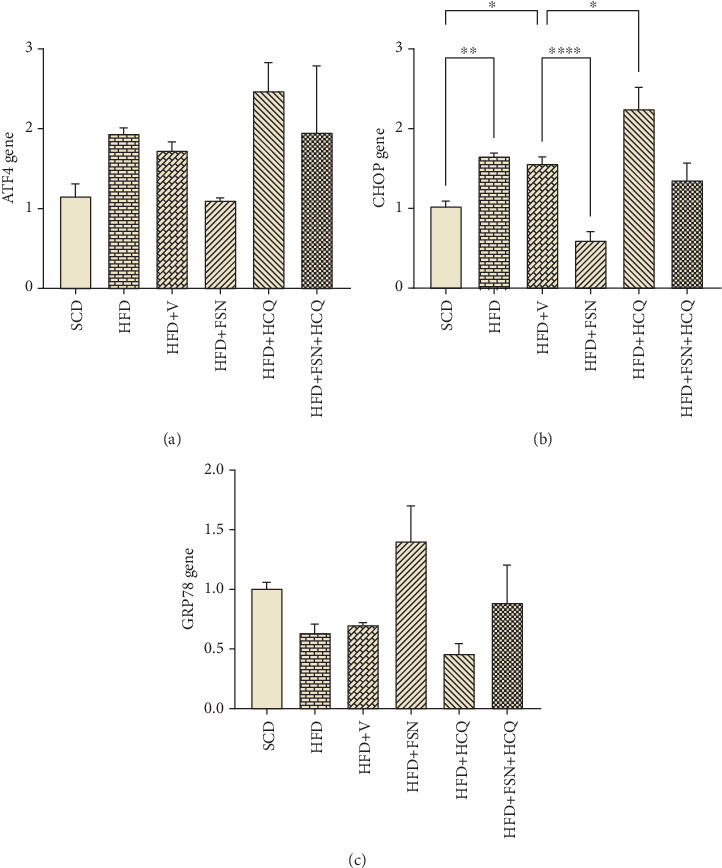
Reinforcement of HFD-induced ER stress by hydroxychloroquine and inhibition by fisetin in mouse pancreas. The mRNA level of (a) ATF4, (b) CHOP, and (c) GRP78 genes was measured by real-time PCR (*n* = 5). Values are presented as means ± SEM. ∗∗∗∗*p* < 0.0001, ∗∗∗*p* < 0.001, ∗∗*p* < 0.01, ∗*p* < 0.05. HFD, high-fat diet; V, vehicle; SCD, standard chow diet; FSN, fisetin; HCQ, hydroxychloroquine; ER, endoplasmic reticulum; ATF4, activating transcription Factor 4; CHOP, C/EBP homologous protein; GRP78, glucose-regulated Protein 78.

To investigate the mechanisms by which HFD, FSN, and HCQ affect ER function in the pancreas of mice, we examined the gene expression of ATF4, in addition to CHOP and GRP78. Following HFD consumption, we observed a modest increase in ATF4 mRNA (~60%) (Figure 3a). Furthermore, HFD (*p* < 0.01) group and HFD + V (*p* < 0.05) groups had a significant rise in CHOP mRNA in compare to SCD (Figure 3b). These changes were further pronounced in the HFD + HCQ group (*p* < 0.05) (Figure 3c). Meanwhile, FSN treatment significantly decreased CHOP expression (*p* < 0.0001) (Figure 3b), moderately lowered the ATF4 response (Figure 3a) and strengthened the levels of GRP78 (Figure 3c). Our findings suggest that HFD promotes the UPR by elevating the expression of ATF4 and CHOP, while HCQ intensifies this effect. Conversely, FSN appears to mitigate UPR by regulating the levels of the GRP78.

### 3.4. Restoration of HFD-Reduced Autophagy in the Pancreas of Mice by FSN

**Figure 4:**
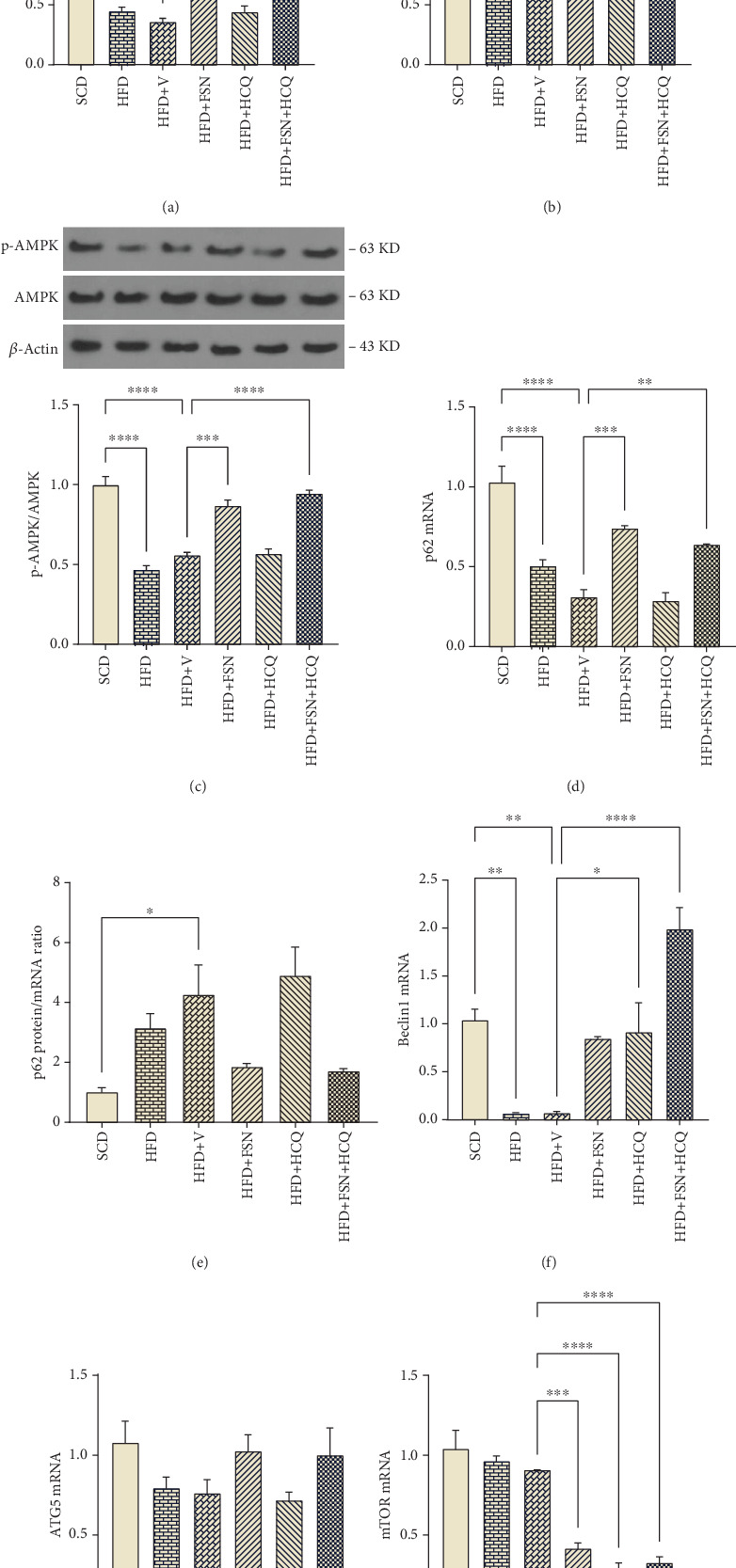
Restoration of HFD-reduced autophagy in the pancreas of mice by fisetin. The results from the western blot of proteins involved in autophagy: (a) LC3II/LC3I, (b) p62, and (c) AMPK phosphorylation in mice (*n* = 3). (d) The expression level of p62 gene, (e) p62 protein/mRNA ratio. The expression levels of (f) Beclin1, (g) ATG5, and (h) mTOR (*n* = 5). Data are presented as mean ± SEM. Values are significantly different between groups as determined using one-way ANOVA. ∗∗∗∗*p* < 0.0001, ∗∗∗*p* < 0.001, ∗∗*p* < 0.01, ∗*p* < 0.05. HFD, high-fat diet; V, vehicle; SCD, standard chow diet; FSN, fisetin; HCQ, hydroxychloroquine; LC3, microtubule-associated Protein 1A/1B light chain 3; p62, Sequestosome 1; AMPK, AMP-activated protein kinase; Beclin1, Beclin 1; ATG5, autophagy-related Protein 5; mTOR, mechanistic target of rapamycin.

To assess the relationship between FSN and autophagy flux, we performed immune blot analysis of p-AMPK as its prompter, along with the LC3II/LC3I ratio and Sequestosome 1 (p62) as common substrates. We also measured the transcription levels of mTOR, BECN1, p62, and ATG5 genes. Additionally, we calculated the protein-to-transcript ratio of p62 to account for variations in its production on overall protein levels. In pancreas tissue lysates of C57BL/6 J mice, the LC3II/LC3I ratio was significantly diminished by HFD (*p* < 0.0001) and HFD + V (*p* < 0.0001) (Figure 4a). Moreover, p62 protein levels in the HFD group were accumulated (*p* < 0.01) (Figure 4b,e), despite a significant decrease in its expression (*p* < 0.0001) (Figure 4d). The p62 protein/mRNA ratio in the HFD + V group was also accumulated (*p* < 0.05) (Figure 4e). Following FSN treatment, the LC3II/LC3I ratio was significantly higher in both HFD + FSN and HFD + FSN + HCQ treatment groups compared to the vehicle group (*p* < 0.0001) (Figure 4a). In these groups, the p62 protein/mRNA ratio decreased to nearly half of that observed in the HFD + V group, although this reduction was not statistically significant. This decrease in the p62 protein/mRNA ratio was partly due to a decline in protein levels (Figure 4b) and largely attributed to a significant increase in mRNA levels in HFD + FSN (*p* < 0.001) and HFD + FSN + HCQ (*p* < 0.01) groups (Figure 4d). In the context of HFD, p-AMPK levels were reduced (*p* < 0.0001) (Figure 4c), which was associated with a significant downregulation of BECN1 (*p* < 0.01) and a ~29% reduction in ATG5 (Figure 4f,g), occurring independently of mTOR activation (Figure 4h). We also assessed autophagic flux using the lysosomal inhibitor HCQ. In the HFD + HCQ and HFD + FSN + HCQ groups, there was no additional buildup in the LC3II (Figure 4a) or p62 (Figure 4e) compared to the HFD + V and HCQ + FSN groups, respectively. HCQ did not affect p-AMPK levels in HFD + HCQ group (Figure 4c) but significantly increased the expression of Beclin1 (*p* < 0.05) (Figure 4f) and decreased mTOR expression (*p* < 0.0001) compared to vehicle group (Figure 4h).

## 4. Discussion

Obesity and metabolic syndrome have been recognized as NAFPD risk factors. A role of NAFPD in the development of “prediabetes” and T2DM has been frequently suggested. Accumulation of fat in pancreatic tissue possibly initiates a vicious cycle of *β*-cell deterioration and further pancreatic fat accumulation [28, 29]. Weight loss decreases pancreatic fat content [30], but few pharmacologic treatments for NAFPD have been evaluated in specifically designed studies. Matheus et al. used diet supplementation with butyrate to inhibit the deleterious effects of HFD intake on metabolic parameters and function of the pancreas and other organs associated with T2DM in a mouse model [31]. In another study, Sahin et al. found that 7,8-dihydroxyflavone ameliorated metabolic abnormalities in the pancreas of male C57BL/6 mice [32]. Likewise, in this study, we utilized FSN and HCQ, individually or in combination, to demonstrate how they act during HFD-induced NAFPD in an animal model. The selection of the dosage of FSN is based on similar studies conducted in the field [33]. HCQ has been utilized in a wide range of doses across various research settings [34, 35]. In our study, considering the prolonged duration of treatment, a low to moderate dose has been selected to ensure safety and efficacy while minimizing potential side effects.

The extent and distribution of fatty buildup in the pancreas vary significantly among various animal species and strains, with ICR and C57BL/6 mice showing much lower levels compared to humans and rats, as well as Syrian golden hamsters [3]. In our research, a 24-week HFD period triggered metabolic syndrome and a chronic pancreatitis which indicated by adipocytes and inflammatory cell infiltration. In a research experiment, a 12-week period of a HFD resulted in a range of pancreatic alterations in male C57BL/6 mice, such as acinar cell damage involving hypertrophy, autophagy, apoptosis, necrosis, and atrophy, as well as vascular damage, interstitial edema, inflammation, fat necrosis, and ductal changes [36]. In another study, 15 weeks of HFD in C57BL/6 was associated with an increase in the number of adipocytes and to a lesser extent of intracellular lipid vesicles in the pancreas [37]. On top of that, another study found no fat infiltration in pancreatic tissue; however, even though fat was not directly observed in the pancreas, *β*-cells still underwent increased apoptosis as a result of HFD [38]. Indeed, insulin resistance in *β*-cells caused by obesity results in reduced expression of genes involved in cell proliferation, leading to a decrease in *β*-cell mass [39]. Here, we showed that FSN along with HCQ can counteract the detrimental impact of overnutrition and obesity on the gradual deterioration of pancreatic tissue caused by fat infiltration.

Ectopic visceral deposition of fat, particularly in the pancreas, is a significant contributor to insulin resistance and metabolic syndrome. Recent research by Elhady et al. revealed that obese children with fatty pancreas were more likely to have metabolic syndrome and insulin resistance [40]. Our own study further demonstrated a relationship between the severity of NAFPD and disturbances in carbohydrate metabolism, as indicated by increased HOMA-IR and disrupted OGTT, while treatment with FSN, HCQ, and a combination of both counteracted these conditions by insulin sensitivity enhancement. This eliminates the need for compensatory hyperinsulinemia which in turn causes *β* cells to be exhausted and progress from this state of prediabetes to diabetes. FSN has been shown to reduce insulin resistance markers and improve glucose tolerance even at lower doses and shorter treatment durations in other studies [41, 42]. It appears that these effects are enhanced when FSN is administered along with HFD prophylactically. In recent studies, there is a consensus regarding the effect of HCQ on systemic insulin sensitivity; however, the results concerning its impact on pancreatic endocrine function vary. One study on mice fed a HFD as a model for hepatic steatosis reported that HCQ improves insulin sensitivity (based on HOMA-IR and HOMA-IS) without affecting *β*-cell function (based on HOMA-*β*) [43]. Nevertheless, more detailed investigations have yielded differing results. For instance, in HFD-fed rats, morphometric analyses indicated that HCQ not only enhances metabolic characteristics but also helps preserve the structure of the Langerhans islets [44]. A clinical study further demonstrated the beneficial effects of HCQ on *β*-cell function, assessed via the Disposition Index, although no significant difference was observed in acute insulin secretion (AIR) [45]. Additionally, the euglycemic clamp test conducted in insulin-resistant adults indicated improved whole-body insulin sensitivity with HCQ treatment, which was accompanied by an increase in adiponectin levels and a reduction in IL-6 levels in the bloodstream [24].

In the event that *β*-cell ER homeostasis is compromised, dysfunction, apoptosis, and diabetes may result. ER stress has been implicated in the pathogenesis of both types of diabetes: T2DM, which is primarily influenced by external factors such as redox imbalance and glucolipotoxicity that affect ER protein folding efficiency [46], and the monogenic forms related to T1DM, where genes directly affect *β*-cell function and ER stress. For example, mutations in the WFS1 gene, which codes for the ER membrane protein Wolframin, cause Wolfram syndrome, a condition linked to diabetes [47]. Therefore, targeting ER stress in *β*-cell has emerged as a potential therapeutic strategy in diabetes, as regulating insulin secretion from *β*-cells could potentially slow down the advancement of a pivotal component of the metabolic syndrome [10]. In our research, we examined the gene expression of ATF4—a key component of one branch of the UPR—as well as CHOP, which serves as an indicator of overall ER stress severity, and GRP78, an ER chaperone that functions upstream of the UPR, and demonstrated that HFD, both independently and particularly when combined with HCQ, induces ER stress in the pancreas. Studies involving CQ and HCQ have yielded divergent results regarding the suppression or induction of the UPR within the ER. For example, it has been shown that CQ can induce ER stress, thereby promoting apoptosis and countering primary effusion lymphoma. Indeed, part of this effect may be attributed to the inhibition of autophagy [48]. In other investigations, HCQ has been reported to reduce ER stress, potentially as a consequence of inflammation inhibition [49, 50]. It is noteworthy that these findings pertain to various disease models across different tissues and involve variable dosages of HCQ, as well as the duration of drug administration and the stage of the disease. It is evident that each of these factors can significantly influence the observed heterogeneity in results. Findings in our study revealed that FSN has the ability to mitigate these HCQ effects in part by GRP78 chaperon magnification. One study demonstrated that FSN alleviates nonalcoholic fatty liver disease development by inhibiting ER stress. This effect was confirmed through the repression of GRP78 and CHOP expression in hepatocytes and hepatic tissue [33]. This is consistent with our results, indicating that FSN can reduce the effects of HFD and HCQ partly through the enhancement of the chaperone GRP78.

In exploring FSN's role in managing ER stress, we observed its potential to enhance autophagy, a function that is hindered by HFD in the context of NAFPD. FSN increased the LC3II/LC3I ratio and activated AMPK phosphorylation, suggesting autophagy induction. Moreover, FSN regulated the transcription of autophagy-related genes, highlighting its potential contribution to mitigating ER stress through autophagy stimulation. Considering that p-AMPK can promote autophagy either directly or indirectly by inhibiting mTOR, it may be concluded that the autophagy boosting with FSN originates from the AMPK/mTOR pathway. Furthermore, the results revealed that, despite stimulating the expression of p62, FSN accelerated the autophagy process, resulting in the reduction of p62 protein as an autophagy substrate. In an investigation of acetaminophen-induced liver injury, the administration of FSN (20 and 80 mg/kg) resulted in increased ATG5 expression and a reduction in liver injury. The absence of this effect in the presence of si-ATG5 and methyl adenine emphasizes the corroborating results regarding FSN's autophagy-inducing properties [51]. Moreover, in a study focused on sepsis-associated encephalopathy in rats, FSN (20 mg/kg, oral) led to increased autophagy (evidenced by elevated LC3-II and decreased p62 levels) in cerebral microvascular endothelial cells. It also enhanced mitophagy (implicating increased expression of Pink1 and Parkin) [52]. The collective evidence from these studies supports our findings that FSN effectively induces autophagy and mitigates stress in various biological settings, highlighting its therapeutic potential in related pathologies.

With HCQ administration, while the expectation was that the LC3II/LC3I ratio would increase, this was not observed. It seems that long-term treatment with HFD strongly inhibits autophagy, making pharmacological or even genetic interventions no more effective in this reduction. As in a study by Undamatla et al., both PARKIN knockout and wild-type mice converged in hepatic steatosis after long-term (20 weeks) of a Western diet, although significant differences were observed between the two groups after short term (6 weeks) [53]. Thus, limitations of our study include the lack of examining autophagic flux at different time points. Moreover, there is inconsistency in studies regarding whether CQ and HCQ can serve as reliable replacements for other forms of late-stage lysosomal inhibitors in in vivo experiments [54]. Furthermore, HCQ in our study influenced on the expression of some genes, suggesting that it has long-term effects on gene expression patterns. Thus, its overall impact on autophagy extends beyond the short-term acute inhibition observed in cell cultures, which is primarily due to the disruption of lysosomal function. Apart from autophagy inhibitory aspect of HCQ, here, we used the combined treatment of it along with FSN, which significantly improved glucose and insulin homeostasis in HFD-fed C57BL/6 mice, compared to either treatment alone. This improvement may be due to the independent effects of HCQ on insulin function, as shown in a randomized trial [45]. However, our study warns against using HCQ alone due to potential adverse effects such as increased ER stress and sudden idiopathic weight loss observed in mice with prolonged treatment, as this side effect cannot be overlooked. Additionally, reported side effects of HCQ further support this caution [55, 56]. Nevertheless, HCQ does also have benefits for managing chronic inflammation in metabolic syndrome. Therefore, it may be advisable to prescribe HCQ alongside a companion treatment like FSN to address these issues.

In our study, we pioneered the use of a combined treatment involving FSN and HCQ for T2DM management, highlighting its potential benefits for the first time. However, it is important to note that our research represents a preliminary investigation in this field, necessitating further studies to clarify various uncertainties that have emerged. Future research should focus on establishing an optimal dosage balance for these two drugs in metabolic disorders to achieve maximum efficacy while minimizing toxicity. Given that both medications have progressed to human studies, clinical trials, with appropriate precautions, could allow for the evaluation of result concordance in a human population. Furthermore, mechanistic studies are essential to comprehensively assess metabolic aspects—from pancreatic function to the responsiveness of insulin target tissues. This could include the use of transgenic animal models to more accurately evaluate the impact of autophagy activity in pancreatic tissue. It is crucial that these studies include sampling at various stages of fat deposition progression to provide clarity on each dimension. Understanding these factors will facilitate more informed decision-making regarding the implementation of this combined treatment in specific clinical settings.

## 5. Conclusion

In conclusion, our study highlights the significant role of FSN and HCQ in mitigating the adverse effects of HFD-induced NAFPD and its progression to T2DM. This research provides new insights into the role of FSN in regulating autophagy and ER stress in the context of NAFPD, which has not been extensively studied before. We demonstrated that these treatments can enhance insulin sensitivity and counteract the detrimental effects of ectopic fat deposition in the pancreas, which contributes to metabolic syndrome. Importantly, while HCQ alone may induce ER stress, FSN exhibits protective effects, potentially through autophagy induction. Given that ER stress significantly contributes to NAFPD development and considering FSN's ability to mitigate ER stress and enhance autophagy, a combination therapy with HCQ may not be essential for effectively managing NAFPD and related glucose metabolism disturbances. Adjusting the FSN dosage alone might achieve comparable metabolic control. Nevertheless, our research offers valuable insights for individuals who require HCQ treatment and are, therefore, at risk of ER stress and its metabolic consequences; in such cases, FSN may serve a protective role.

## Data Availability

The data that support the findings of this study are available from the corresponding authors upon reasonable request.

## Nomenclature

ATF4: activating transcription Factor 4
AUC: area under the curve
ATG5: autophagy-related Protein 5
ATG6/Beclin-1: autophagy-related Protein 6
CHOP: C/EBP homologous protein
ER: endoplasmic reticulum
FSN: fisetin
HCQ: hydroxychloroquine
mTOR: mechanistic target of rapamycin
NAFPD: nonalcoholic fatty pancreatic disease
p62/SQSTM1: Sequestosome 1
SCD: standard chow diet
T2DM: Type 2 diabetes
UPR: unfolded protein response
GRP78: 78 kDa glucose-regulated protein
V: vehicle (in experiments)
HFD: high-fat diet
