# Supplementary material for: Optimized Effects of Fisetin and Hydroxychloroquine on ER Stress and Autophagy in Nonalcoholic Fatty Pancreas Disease in Mice
Source: J Diabetes Res. 2025 Apr 14;2025:2795127. doi: 10.1155/jdr/2795127 (PMC12011465; doi:10.1155/jdr/2795127)
Supplement: Supporting Information — Additional supporting information can be found online in the Supporting Information section. Table S1: Primer sequences used in this study. [file 2795127.f1.docx]

**Supplementary Table 1**

Primers sequences used in this study.

| **Reverse** | **Forward** | **Primer** |
| --- | --- | --- |
| **5ʹ- ATGGAGCCACCGATCCACA -3ʹ** | **5ʹ-CATCCGTAAAGACCTCTATGCCAAC -3ʹ** | **β-Actin** |
| **5ʹ- GGAGGAGACACGAAGCAGACT -3ʹ** | **5ʹ- ACAACACTGACCTGGACACTT -3ʹ** | **GRP78** |
| **5ʹ- ACTGTGGCGTTAGAGATCGT -3ʹ** | **5ʹ- CGGCTGGTCGTCAACCTAT -3ʹ** | **ATF4** |
| **5ʹ- GGTGCCCCCAATTTCATCT -3ʹ** | **5ʹ- CCACCACACCTGAAAGCAGAA -3ʹ** | **CHOP** |
| **5ʹ- AAGGTGGCATTGAAGACATT -3ʹ** | **5ʹ- AGCGGGAGTATAGTGAGTTT -3ʹ** | **Beclin-1** |
| **5ʹ- ACGAAGGCTGGGTTCATGC -3ʹ** | **5ʹ- GTTTGTGGCTCTGAATGACCA -3ʹ** | **MTOR** |
| **5ʹ- TTCTGGGGTAGTGGGTGTCA -3ʹ** | **5ʹ- GAATGTGGGGGAGAGTGTGG -3ʹ** | **SQSTM1/p62** |
| **5ʹ- TCCAAGGAAGAGCTGAACTTGA -3ʹ** | **5ʹ- AAGGCACACCCCTGAAATGG -3ʹ** | **ATG5** |
